# Supplementary material for: Haemophilus influenzae Meningitis Direct Diagnosis by Metagenomic Next-Generation Sequencing: A Case Report
Source: Pathogens. 2021 Apr 12;10(4):461. doi: 10.3390/pathogens10040461 (PMC8069228; doi:10.3390/pathogens10040461)
Supplement: Supplementary file 1 [file pathogens-10-00461-s001.pdf]

File S1: Antibiogram analysis

1) In silico data analysis.

Antimicrobial	Class	WGS-predicted phenotype	Match	Genetic background
**Ceftriaxone**	beta-lactam	No resistance	0	
**ampicillin+clavulanic acid**	beta-lactam	No resistance	0	
**piperacillin+clavulanic acid**	beta-lactam	No resistance	0	
**cephalothin**	beta-lactam	No resistance	0	
**ticarcillin+clavulanic acid**	beta-lactam	No resistance	0	
**cefoxitin**	beta-lactam	No resistance	0	
**ampicillin**	beta-lactam	No resistance	0	
**ceftazidime**	beta-lactam	No resistance	0	
**amoxicillin+clavulanic acid**	beta-lactam	No resistance	0	
**piperacillin**	beta-lactam	No resistance	0	
**cefepime**	beta-lactam	No resistance	0	
**penicillin**	beta-lactam	No resistance	0	
**ertapenem**	beta-lactam	No resistance	0	
**ceftazidime+avibactam**	beta-lactam	No resistance	0	
**imipenem**	beta-lactam	No resistance	0	
**cephalotin**	beta-lactam	No resistance	0	
**meropenem**	beta-lactam	No resistance	0	
**aztreonam**	beta-lactam	No resistance	0	
**temocillin**	beta-lactam	No resistance	0	
**cefotaxime+clavulanic acid**	beta-lactam	No resistance	0	
**cefixime**	beta-lactam	No resistance	0	
**amoxicillin**	beta-lactam	No resistance	0	
**ticarcillin**	beta-lactam	No resistance	0	
**unknown beta-lactam**	beta-lactam	No resistance	0	
**piperacillin+tazobactam**	beta-lactam	No resistance	0	
**cefotaxime**	beta-lactam	No resistance	0	
**sulfamethoxazole**	folate pathway antagonist	No resistance	0	
**trimethoprim**	folate pathway antagonist	No resistance	0	
**linezolid**	oxazolidinone	No resistance	0	
**lincomycin**	lincosamide	No resistance	0	
**clindamycin**	lincosamide	No resistance	0	
**chloramphenicol**	phenicol	No resistance	0	
**florfenicol**	phenicol	No resistance	0	
**unknown phenicol**	phenicol	No resistance	0	
**spectinomycin**	aminocyclitol	No resistance	0	
**tigecycline**	tetracycline	No resistance	0	
**doxycycline**	tetracycline	No resistance	0	
**tetracycline**	tetracycline	No resistance	0	
**unknown tetracycline**	tetracycline	No resistance	0	
**minocycline**	tetracycline	No resistance	0	
**colistin**	polymyxin	No resistance	0	
**fusidic acid**	steroid antibacterial	No resistance	0	
**mupirocin**	pseudomonic acid	No resistance	0	

**tiamulin**	pleuromutilin	No resistance	0
**nalidixic acid**	fluoroquinolone	No resistance	0
**unknown fluoroquinolone**	fluoroquinolone	No resistance	0
**ciprofloxacin**	fluoroquinolone	No resistance	0
**fluoroquinolone**	under_development	No resistance	0
**virginiamycin m**	streptogramin a	No resistance	0
**dalfopristin**	streptogramin a	No resistance	0
**pristinamycin iia**	streptogramin a	No resistance	0
**quinupristin+dalfopristin**	streptogramin a	No resistance	0
**telithromycin**	macrolide	No resistance	0
**tylosin**	macrolide	No resistance	0
**oleandomycin**	macrolide	No resistance	0
**azithromycin**	macrolide	No resistance	0
**carbomycin**	macrolide	No resistance	0
**spiramycin**	macrolide	No resistance	0
**unknown macrolide**	macrolide	No resistance	0
**erythromycin**	macrolide	No resistance	0
**teicoplanin**	glycopeptide	No resistance	0
**vancomycin**	glycopeptide	No resistance	0
**unknown aminoglycoside**	aminoglycoside	No resistance	0
**butirosin**	aminoglycoside	No resistance	0
**streptomycin**	aminoglycoside	No resistance	0
**fortimicin**	aminoglycoside	No resistance	0
**apramycin**	aminoglycoside	No resistance	0
**kanamycin**	aminoglycoside	No resistance	0
**paromomycin**	aminoglycoside	No resistance	0
**amikacin**	aminoglycoside	No resistance	0
**gentamicin**	aminoglycoside	No resistance	0
**hygromycin**	aminoglycoside	No resistance	0
**astromicin**	aminoglycoside	No resistance	0
**lividomycin**	aminoglycoside	No resistance	0
**netilmicin**	aminoglycoside	No resistance	0
**isepamicin**	aminoglycoside	No resistance	0
**tobramycin**	aminoglycoside	No resistance	0
**neomycin**	aminoglycoside	No resistance	0
**sisomicin**	aminoglycoside	No resistance	0
**dibekacin**	aminoglycoside	No resistance	0
**arbekacin**	aminoglycoside	No resistance	0
**kasugamycin**	aminoglycoside	No resistance	0
**butiromycin**	aminoglycoside	No resistance	0
**ribostamycin**	aminoglycoside	No resistance	0
**metronidazole**	nitroimidazole	No resistance	0
**fosfomycin**	fosfomycin	No resistance	0
**quinupristin**	streptogramin b	No resistance	0
**virginiamycin s**	streptogramin b	No resistance	0
**pristinamycin ia**	streptogramin b	No resistance	0
**unknown rifamycin**	rifamycin	No resistance	0
**rifampicin**	rifamycin	No resistance	0

2) Routine antibiogram analysis

ANTIBIOTIC TEST	VALUE	STAT
**AMOXICILLINE E-TEST**	0.750 mg/L	Sensitive
**AUGMENTIN**	<=1 mg/L	Sensitive
**CEFTRIAXONE E-TEST**	0.016 mg/L	Sensitive
**GENTAMYCINE**	2 mg/L	Sensitive
**NALIDIXIC ACIDE**	NA	Sensitive
